# Design of Experiment for Optimizing Microencapsulation by the Solvent Evaporation Technique

**DOI:** 10.3390/polym16010111

**Published:** 2023-12-29

**Authors:** Mónica V. Loureiro, António Aguiar, Rui G. dos Santos, João C. Bordado, Isabel Pinho, Ana C. Marques

**Affiliations:** 1 CERENA—Centro de Recursos Naturais e Ambiente, Departamento de Engenharia Química, Instituto Superior Técnico, Universidade de Lisboa, Avenida Rovisco Pais, 1049-001 Lisbon, Portugal; antonio.luis.aguiar@tecnico.ulisboa.pt (A.A.); rui.galhano@tecnico.ulisboa.pt (R.G.d.S.); jcbordado@tecnico.ulisboa.pt (J.C.B.); 2 CIPADE—Indústria e Investigação de Produtos Adesivos, SA. Av. Primeiro de Maio 121, 3700-227 São João da Madeira, Portugal; isabel.pinho@cipade.com

**Keywords:** microencapsulation, DoE, polycaprolactone, isocyanate, adhesives

## Abstract

We employed microemulsion combined with the solvent evaporation technique to produce biodegradable polycaprolactone (PCL) MCs, containing encapsulated isophorone diisocyanate (IPDI), to act as crosslinkers in high-performance adhesive formulations. The MC production process was optimized by applying a design of experiment (DoE) statistical approach, aimed at decreasing the MCs’ average size. For that, three different factors were considered, namely the concentration of two emulsifiers, polyvinyl alcohol (PVA) and gum arabic (GA); and the oil-to-water phase ratio of the emulsion. The significance of each factor was evaluated, and a predictive model was developed. We were able to decrease the average MC size from 326 μm to 70 µm, maintaining a high encapsulation yield of approximately 60% of the MCs’ weight, and a very satisfactory shelf life. The MCs’ average size optimization enabled us to obtain an improved distributive and dispersive mixture of isocyanate-loaded MCs at the adhesive bond. The MCs’ suitability as crosslinkers for footwear adhesives was assessed following industry standards. Peel tests revealed peel strength values above the minimum required for casual footwear, while the creep test results indicated an effective crosslinking of the adhesive. These results confirm the ability of the MCs to release IPDI during the adhesion process and act as crosslinkers for new adhesive formulations.

## 1. Introduction

Polyurethane (PU) and polychloroprene (PCP) adhesives are currently supplied as two-component (2K) adhesive formulations composed of a pre-polymer and a diisocyanate or polyisocyanate as crosslinker [1,2]. The incorporation of isocyanate crosslinkers is essential to providing a strong and long-lasting adhesive formulation [1,2]. However, isocyanates have a high toxicity, which becomes a primary concern during the adhesive application. Isocyanates are powerful irritants, mainly affecting the mucous membranes of the eyes, respiratory, and gastrointestinal tracts as well as causing skin inflammation when in direct contact, and prolonged exposure can lead to severe asthma attacks and even death [3]. To avoid health risks, in February 2020, the REACH regulation approved the European Commission’s proposal regarding restriction on diisocyanates, which prohibits the marketing of these chemicals alone and as constituents of other substances or mixtures when in concentrations greater than 0.1% by weight [4]. However, the use of isocyanates at lower concentrations does not guarantee an adhesive with the same final properties. To tackle this, our research group proposed to develop new PU and PCP adhesive formulations for the footwear industry, in which the crosslinkers would be polymeric microcapsules (MCs) containing isocyanate species [5,6,7,8,9]. By protecting the isocyanate inside polymeric MCs, direct contact with the operator is avoided. The isocyanate is only to be released during adhesive joint preparation, either by melting, due to the effect of temperature (70 °C) and/or by breaking, due to the effect of pressure (4 bar) applied during adhesive joint manufacturing.

We previously reported on the encapsulation of isocyanate species with the commonly used interfacial polymerization technique, in polyurethane/polyurea (PU/PUa) shelled MCs, and the solvent evaporation method, in biodegradable shelled MCs [5,6,7,8,9]. Isocyanate microencapsulation is typically reported to be achieved using an oil-in-water (O/W) micro-emulsion system combined with the interfacial polymerization technique [10,11,12,13,14,15,16]. It is a simple, reliable, well-studied, and low-cost process to produce isocyanate-loaded MCs. Several types of MCs have been produced using the referred technique mainly with a PU, PU/PUa and its combination with poly(urea-formaldehyde) (PUF) shell. The microencapsulation of isocyanate dates from 2008, with the encapsulation of monomeric isophorone diisocyanate (IPDI) [10], resourcing to a 2,4-TDI prepolymer, as a higher reactivity isocyanate, for the shell formation, and 1,4-butanediol as an extra active hydrogen (H) source and chain extender. Other isocyanates used for the shell formation, as well as other active H sources have been reported for the synthesis of IPDI loaded MCs, obtained using the referred technique [11,12,13,14,15,16]. Despite their advantages, the obtained MCs are not temperature responsive, have a poor shelf life and their shell exhibits none or low biodegradability.

The high reactivity of isocyanates, along with their typical high viscosity, makes their encapsulation a challenge. The solvent evaporation method has shown to be a simple and reliable method to produce biodegradable shelled MCs, with a prolonged shelf life and high loads of encapsulated isocyanate [6,9]. Despite the favorable characteristics of the previously reported PCL MCs, their large size, of average 326 ± 157 µm, and wide size distribution, ranging from ca. 75 to 825 µm, reveal some issues for the current adhesive applications [6]. Large-sized MCs lead to a heterogeneous distribution of the crosslinker in the adhesive layer and cause a disruption effect in the adhesive bondline, worsening the performance of the adhesive formulation. On the other hand, when the MCs are too small, a substantial quantity of them is required to ensure effective adhesive crosslinking. Furthermore, smaller MCs exhibit increased resistance to pressure-induced breakage. A DoE tool was used as a statistical approach to optimize the MC production process aimed at optimizing the MCs’ average size. This method has several advantages over the typical “one factor at a time” (OFAT) method. OFAT encompasses the iterative experimentation of one factor by fixing all process factors except the one that is under optimization [17,18]. Adding to the inefficiency and time-consuming process, it is often inaccurate as an optimization technique for chemical processes, as there are no considerations for synergistic effects. It is a linear experimental procedure, and chemical reaction outputs usually have nonlinear responses [6,18,19,20]. Therefore, it is possible to deduce the individual impact of each factor on the reaction, but it is not possible to draw conclusions regarding their mutual influence on one another. [21,22]. As an alternative, the use of statistical or physical modeling might be considered [18]. DoE is used to systematically address cause and effect relations between the parameters in study and experimental outputs, enabling to build a model to mathematically predict an output [17,21,22,23,24]. The use of DoE was previously reported for the optimization of MCs obtained using several techniques, such as spray-drying, phase separation, solvent evaporation and in situ and interfacial polymerization [25,26,27,28,29,30]. Lock et al. reported on the development of a model to predict the internal architecture and mean particle diameters of gas-filled polymeric MCs, produced using phase separation [26]. The authors considered concentration of emulsifier, stirring speed, and emulsion dilution as factors. It was concluded that stirring speed was the most significant factor affecting the MCs obtained using the referred technique, with a higher speed leading to smaller MCs. This was also observed by Carvalho et al. when applying a DoE approach to reach a conclusion on the effects of pH conditions and stirring speed on poly(urea-formaldehyde) MC size and shell thickness obtained using in situ polymerization [27]. Regarding the use of DoE for the study of MC production using the solvent evaporation technique, Grandhi et al. reported on the optimization of the scale-up process to produce Eudragit RSPO microspheres for the entrapment of lovastatin, considering the processing temperature and the viscosity of the polymer phase as factors. The authors concluded that MCs with a smoother and more uniform surface were formed with higher viscosities of the polymeric phase [28]. Rai et al. reported on the optimization of MCs composed of a Eudragit S100 shell produced via the solvent evaporation technique, using a water-in-oil-in-water (W/O/W) emulsion system. To optimize the MCs’ size and drug load, the authors considered drug-to-polymer ratio, surfactant concentration, and stirring speed as factors, obtaining smaller particles using a lower polymer concentration, a higher surfactant concentration, and a higher stirring speed [29]. It is notable that the encapsulation content, the emulsion system, and the polymer used for the shell formation differ from those reported in our work. Publications regarding the use of a DoE statistical tool for the optimization of polymeric MCs containing encapsulated isocyanates are very scarce. Javidi et al. reported on the use of DoE in applying a response surface methodology (RSM), with a central composite design (CCD), for the optimization of MCs containing isocyanate species, obtained via interfacial polymerization [30]. For that, stirring speed, isocyanate content and synthesis duration were considered as variables and the MCs’ size, shell thickness, microencapsulation efficiency and core content were evaluated as responses. The authors concluded that the stirring speed is the factor that most impacts the MCs’ size, core content, and encapsulation efficiency.

In this work, we report on the use of a DoE tool as a statistical approach to optimize the average size of biodegradable PCL MCs containing IPDI, obtained via the solvent evaporation technique. The statistical importance of three process parameters known to influence emulsion stability, namely polyvinyl alcohol (PVA) concentration, gum arabic (GA) concentration, and oil (O) to water (W) phase volume of the emulsion, were evaluated in terms of their effect on the MCs’ morphology. To the best of our knowledge, the use of DoE to optimize the production of isocyanate loaded MCs, obtained via the solvent evaporation technique, is not yet reported. The optimized MCs were posteriorly validated as crosslinkers for PU and PCP adhesives, following industrial standards, and the isocyanate’s concentration was optimized in the new adhesive formulation.

## 2. Materials and Methods

### 2.1. Materials

The shell-forming polymer, PCL, with an average molecular weight (MW) of 45,000 Da, was supplied by Sigma-Aldrich (St. Louis, MO, USA). The isocyanate to be encapsulated, IPDI, with the commercial name of Desmodur I, was purchased from Covestro AG (Leverkusen, Germany). The emulsifiers, gum arabic (GA) and poly(vinyl alcohol) (PVA) (98–99% hydrolyzed, with medium MW, 57,000–67,000 Da), were purchased from Fisher Chemical (Porto Salvo, Portugal) and Alfa Aesar (Haverhill, MA, USA), respectively. The Ciprene^®^2000 and Plastik^®^6275 adhesive pre-polymers, as well as the respective crosslinkers, Suprasec^®^ 2234 and Ciprene^®^ 2000, here used as benchmarks, were kindly by CIPADE, S.A (São João da Madeira, Portugal). All the chemicals were used as received, without further purification.

### 2.2. Method

The MC production process involves the solvent evaporation technique in combination with a microemulsion system, identical to that previously reported by Loureiro et al. [6].

Briefly, the IPDI to be encapsulated is mixed with a solution composed of PCL used for the MCs’ shell, dissolved in DCM. This makes the O phase of the emulsion, which is posteriorly dispersed in the W phase containing PVA and GA as emulsifiers. The obtained emulsion is placed under mechanical stirring, at room temperature for the time necessary to reach a complete evaporation of the organic solvent. As the evaporation proceeds, the polymer precipitates around the O phase droplets, forming the MCs. The initial emulsion stability and the MCs’ maturity follow during the MC fabrication process via optical microscopy. The MCs are washed with water and collected via using a vacuum-assisted filtration process.

There were slight modifications to the process parameters, in particular those which were considered independent variables, i.e., the GA and PVA concentrations, as well as the O-to-W phase ratio of the emulsion. The values employed for the referred variables were made to vary according to the experiments proposed by the DoE software, MODDE^®^ Pro 13 (Umetrics, Umeå, Sweden), for statistical analysis.

#### 2.2.1. Characterization

The MCs’ morphology was characterized by optical microscopy and scanning electron microscopy (SEM), and their chemical composition by Fourier transform infrared spectroscopy (FTIR) with attenuated total reflectance (ATR) accessory, thermogravimetric analysis (TGA), and first-derivative curves (DTG).

The adhesives, containing the MCs as crosslinkers, were evaluated in industrial facilities via peel strength tests and temperature resistance tests (creep test).

Optical Microscopy. A Krüss MSZ 5600 optical microscope (Krüss, Hamburg, Germany) was used to evaluate the emulsion stability, droplets’ size, and the MCs’ shell maturity during the MCs’ fabrication.

Scanning Electron Microscopy (SEM). The morphology, size distribution, and shell thickness of the MCs were assessed through SEM, using a field emission gun scanning electron microscope FEG-SEM, JEOL JSM7001F (JEOL, Tokyo, Japan), operating in a range of 10–15 kV at a working distance of 10 mm. The photomicrographs were obtained in secondary electron mode. The samples were immobilized using a conductive double-sided adhesive carbon tape in a sample holder and coated with a conductive 15 nm layer of gold–palladium thin film, through sputtering, using a Quorum Technologies sputter coater, model Q150T ES (Laughton, UK). The photomicrographs were used to evaluate the MCs’ average diameter, size distribution, and shell thickness using Fiji software (ImageJ version 1.53c), on a sample of 200 MCs [31]. Debris and non-spherical MCs were discarded in the calculations.

Fourier Transform Infrared Spectroscopy (FTIR). FTIR was used to confirm the isocyanate encapsulation and to assess the chemical structure of the MCs’ shells. A Spectrum Two from PerkinElmer (Waltham, MA, USA) equipped with an attenuated total reflection (ATR) UATR Two accessory was used. The spectra were acquired at room temperature by averaging 8 scans at a resolution of 4 cm^−1^.

Equation (1) was used to determine the relative encapsulation yield (Y value) of the MCs, which represents an indirect measurement of the isocyanate encapsulation efficiency. For that purpose, the area of the peak regarding the isocyanate NCO group, at 2260 cm^−1^, and the peak at 1720 cm^−1^, correlated to the PCL carbonyl group, were considered.

(1) $$Y=\frac{{Area}_{NCO\left(2260{\;cm}^{-1}\right)}}{{Area}_{Shell\left(1720{\;cm}^{-1}\right)}}$$

where Y is considered the relative encapsulation yield, ${Area}_{NCO\left(2260{\;cm}^{-1}\right)}$ is the area of the isocyanate NCO peak, and ${Area}_{Shell\left(1720{\;cm}^{-1}\right)}$ is the area of the PCL carbonyl stretching peak.

Thermogravimetric Analysis (TGA). The thermograms and respective derivative curves (DTG) were used to quantify the encapsulated isocyanate and conclude regarding the MCs’ shell composition, aimed to corroborating the FTIR analysis results. TGA was performed using a Hitachi STA 7200 Thermal Analysis System (Hitachi, Ltd., Tokyo, Japan) under a controlled nitrogen atmosphere with a flow of 200 mL/min, at a temperature increase rate of 10 °C/min, from 35 to 600 °C.

Peeling strength test. The peeling strength test was performed to measure the adhesive strength of the bond. The strength was calculated during the test by dividing the average force by the unit width of the bonded samples. This test was performed at the CIPADE S.A. facilities using a tensile testing machine and following the ISO 20344:5.2 norm [32], at a constant speed of 1 cm/min and an angle of 180°.

The specimens used for the testing were composed of two Neolite substrates, an artificial substitute for leather which is widely used in the footwear industry, with 13 cm × 3 cm, glued together in an area of 10 cm × 3 cm. Two different pre-polymers were tested for the development of the new adhesive formulation, namely Ciprene^®^2000, a PCP pre-polymer, and Plastik^®^ 6275, a PU pre-polymer. The substrates were subjected to mechanical carding prior to the joint preparation, and for the ones prepared with Plastik^®^ 6275, an additional chemical treatment of halogenation, with 2190 Halinov (CIPADE S.A., São João da Madeira, Portugal), was also necessary. The adhesive formulation, composed of the pre-polymer and crosslinker, which were previously mixed together, was applied to both substrates with a brush and allowed to dry for 15 min at room temperature. Afterward, the substrates prepared with Ciprene^®^ 2000 are pressed together at 4 bar for 10 s, while the ones prepared with Plastik^®^ 6275 required an additional reactivation step processed of infared (IR) radiation at 70 °C for 6 s, using an IR heat activator. The adhesive joints were stored for 7 days in standard conditions (23 °C, 50% RH) to ensure the complete cure of the adhesive.

Temperature Resistance Test (creep test). The temperature resistance test measures the adhesive temperature resistance, which is a manifestation of the adhesive crosslinking, when submitted to constant force. This test will be herein referred to as the creep test. The test was performed at the CIPADE S.A. facilities, using a climatic chamber heat activator from Aralab (Rio de Mouro, Portugal). For this, one of the unbonded ends of the specimen was fixed on the cabinet of the oven, and the other unbonded end was loaded with a weight of 300 g. The temperature resistance test was performed in a controlled environment at a starting temperature of 60 °C, which the samples were subjected for 2 h. After this period, the displacement, in centimeters (cm), was measured. For the samples that did not open completely, the same procedure was repeated at 70 °C and, subsequently, at 80 and 90 °C. A lower displacement represents a better adhesive joint resistance to temperature and, consequently, a better reticulation.

#### 2.2.2. Experimental Design

A DoE approach was employed to optimize the MCs’ morphology, in particular the average MC size. For the DoE the MODDE^®^ Pro 13 software it used and the RSM method with a CCD design was applied, in particular the central composite face design (CCF). Table 1 lists the chosen independent variables and respective ranges under study.

The CCF consisted of three main factors with three levels each, resulting in 14 experiments and 3 replicates of the experimental runs N15, 16, and 17, with their values listed in Table 2. The run order of the experiments was randomized, to prevent any extraneous factors from affecting the results.

A multiple linear regression was used to fit the experimental data, and the following second-order polynomial equation, Equation (2), was used to build a model to describe and predict the outcome responses to the factor’s variations:

(2) $$Y=\beta_{0}+\sum_{i=1}^{3}\beta_{i}x_{i}+\sum_{i=1}^{3}{\beta_{ii}x}_{i}^{2}+\sum_{i=i}^{2}\sum_{j=i+1}^{3}\beta_{ij}x_{i}x_{j}$$

In Equation (2), the variable “Y” represents the response (average MC size, µm), x_i_ and x_j_ the independent variables and β_0_, β_i_, β_ii_, and β_ij_ the intercept, linear, quadratic, and interaction coefficients, respectively [33,34].

## 3. Results and Discussion

Our research group has been working on developing and optimizing the encapsulation of reactive isocyanate in biodegradable shelled MCs using the solvent evaporation technique [6,9]. Previously reported PCL MCs exhibited a relatively high encapsulation load and stability over a period of 3 months and have proved to be efficient adhesive crosslinkers in preliminary laboratory tests [6]. PCL has a low melting point of 60 °C, low melted viscosity, and low glass transition temperature, of about −60 °C. These features enable MCs with a PCL shell to release their content not only through breaking but also through the melting of their shell during adhesive joint preparation. Despite the favorable characteristics of the resulting MCs, their average size of 326 μm and a wide size distribution ranging from 75 to nearly 900 µm was found to be inadequate for adhesive joints at the industrial level.

With the aim of decreasing the MCs’ size, a DoE method was applied, considering three critical and independent variables known to impact emulsion stability: the volume of the O phase in the overall emulsion system and the concentrations of PVA and GA in the emulsion’s W phase. With the solvent evaporation technique, the size and stability of the emulsion droplets are closely correlated with the size of the final MCs, as they are formed via polymer precipitation at the emulsion droplets’ interface. The volume of the dispersed phase in the emulsion, which corresponds to the O phase, has been reported to have a correlation with the size of the emulsion droplets [35,36]. PVA and GA have been used in the MC fabrication process as emulsifiers [6,37,38,39,40]. PVA is a rheology modifier, intended to increase the viscosity of the aqueous solutions, which is known to reduce the tendency toward collisions between the O droplets as well as their sedimentation [39,40], while GA is a branched polysaccharide, used in O/W emulsion stabilization to form a protective film around emulsion droplets, which creates a steric barrier avoiding coalescence [37,38,41]. The optimum range for the average MC size was defined to be between 70 μm and 80 µm, although MCs with an average diameter of 100 µm are still fit for this application. This value was selected considering the performances of the MCs in the adhesive formulations.

For the DoE approach, and according to the CCF design, seventeen experiments were carried out randomly under different conditions of PVA (2 to 4 wt%) and GA (0 to 3.5 wt%) concentrations and O-to-W phase ratios (28 to 39 wt%), with the obtained experimental results, regarding the MCs’ size and encapsulated content of the as-prepared and aged MCs, reported on Table 3. It should be noted that run N17 was deemed an outlier, and thus, only 16 runs were considered for the model development.

**Figure 1:**
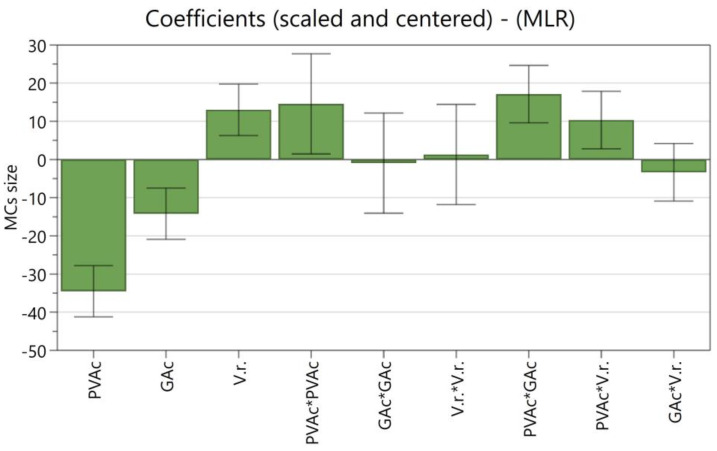
The coefficient plot for the DoE saturated model, showing the influence of individual and squared terms (where * represents multiplication) on the MCs’ average size (N = 16; R^2^ = 0.978; Q^2^ = 0.734; confidence = 0.95).

**Figure 2:**
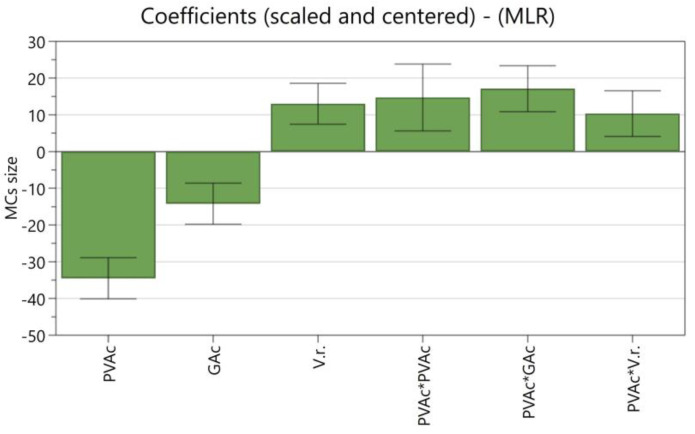
The coefficient plot for the DoE model, considering the significant terms, showing the influence of individual and squared terms (where * represents multiplication) on the MCs average size (N = 16; R^2^ = 0.973; Q^2^ = 0.867; confidence = 0.95).

Figure 1 shows the coefficient plot, in which the size of the bars indicates the magnitude of the effect and the error bar the 95% confidence interval. All the terms are scaled and centered, allowing for the comparison of factors with different units. MODDE uses a saturated model, by which all terms, including all interactions and squared terms, are included, which leads to high R^2^ but low Q^2^ values, as non-significant terms are also considered. Significant terms are those which have a magnitude far from zero and an uncertainty level that does not cross y = 0 [17]. R^2^ and Q^2^ represent how the predictive model fits the experimental data and its accuracy in predicting new data, respectively. Ideally, the model should have a high R^2^, which enables it to explain the dataset, and a high Q^2^ to interpolate new data points accurately [17,42]. To obtain a high Q^2^, non-significant terms must not be considered. Three terms were removed, increasing the Q^2^ from 0.734 to 0.867. The new coefficient plot is presented in Figure 2.

The most significant factor showing a linear effect on the MCs’ average size is the PVA concentration, with its increase leading to a decrease in the MCs’ size. Additionally, PVA concentration is the only factor with a significant quadratic relationship with the MCs’ average size as well as relevant interactions with the other parameters. The PVA concentration and its correlations with the other parameters have a higher impact on the average MC size, while the GA concentration and the O phase volume in the emulsion were found to have a lower statistical significance.

A second-order analysis of the resulting experimental design showed a good correlation with the experimental data, with a R^2^ of 0.973 and a Q^2^ of 0.867, showd in Table 4.

The equation for the model, Equation (3), which enables the prediction of the values of the variables, was computed based upon the correlation coefficients and their effect on the MCs’ average size.

(3) $$Y\left(µm\right)=85.395-34.5122x_{1}-14.2052x_{2}+13.0118x_{3}+14.7212x_{1}^{2}+17.1165{x_{1}x}_{2}+10.329x_{1}x_{3}$$

For Equation (3), the variable Y is the average MC size (μm), x_1_ is the PVA concentration in the W phase, x_2_ the GA concentration in the W phase, and x_3_ the O phase volume in the emulsion system.

**Figure 3:**
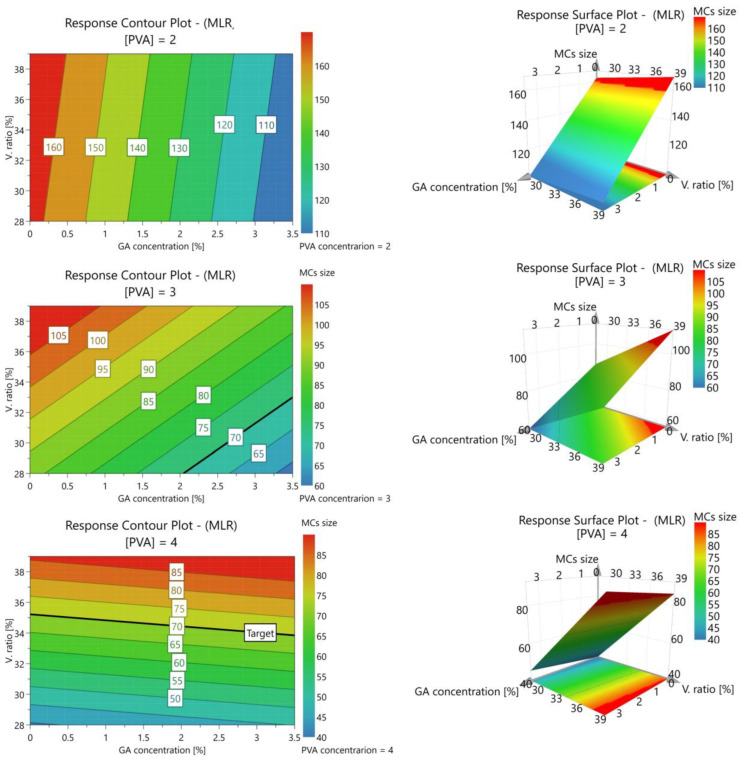
Contour plots of the response (MC size, µm) to the variation of GA concentration (*w*/*w* %) vs. volumetric ratio between the O and W phases (*v*/*v* %) for different PVA concentrations (**left**) and the respective 3-D surface plot (**right**).

Figure 3 shows the response contour and surface plots from the modeling of the interactive effect of the variables in study, on the average MC size. The counter plots show the minimum MCs’ diameters represented in blue and the larger represented in red. By increasing the PVA concentration from 2 wt% to 3 wt%, the shift of the MCs’ size range is clearly visible, covered by the contour plot, to smaller values, confirming the effect of this variable. For 2 and 3 wt% of PVA in the W phase, there is a tendency for smaller MCs to be obtained by increasing the concentrations of GA and/or lowering the concentrations of the O phase in the emulsion. For a PVA concentration of 4 wt%, an increase in the MCs diameter with the increase of the GA concentration is observed. High concentrations of both PVA and GA can lead to an excessive increase in the emulsion viscosity, requiring a higher mechanical energy input to form smaller droplets during the emulsification process [35]. The necessary mechanical energy input can be provided by an extended emulsification time or an increase in the emulsification rate. Regarding the emulsion phase ratios, a correlation between an increase in droplet size with an increase in the dispersed phase volume was already reported [43]. This phenomenon can be correlated with a lack of emulsion stability causing coalescence due to an insufficient concentration of emulsifier to cover the O droplets [36,44,45]. An increase in the O phase volume is also associated with a higher emulsion viscosity.

Two additional validation experiments were conducted, aiming at an average diameter of 70 μm, with the variable’s values given in Table 5, along with the predicted and experimental outcomes. These runs, N18 and N19, fall within the predicted values using a confidence level of 95%, which validates the model. The obtained equation can therefore be considered valid to predict the average MC sizes as a function of GA and PVA concentration and O phase volume in the emulsion.

**Figure 4:**
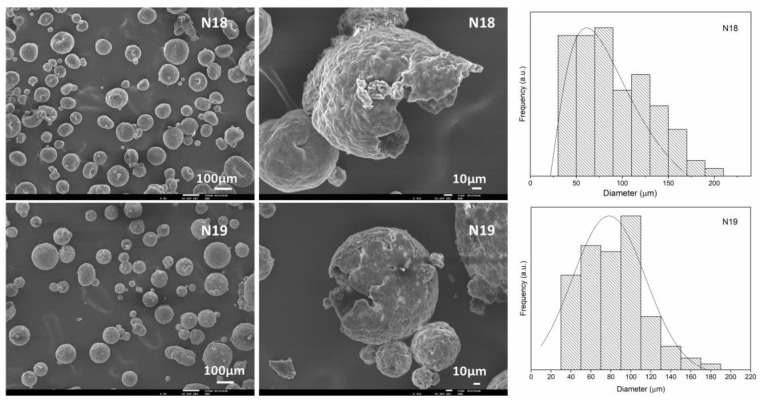
SEM images of the MCs obtained in the validation runs N18 and N19.

These MCs (N18 and N19) are loose and spherical with a core-shell morphology (Figure 4). Both syntheses resulted in identical size distribution ranges, with the N18 MCs having the size distribution peak shifted to smaller sizes. Both size distributions are significantly narrower compared to the previously reported PCL MCs [6], which ranged from 75 to nearly 900 µm.

**Figure 5:**
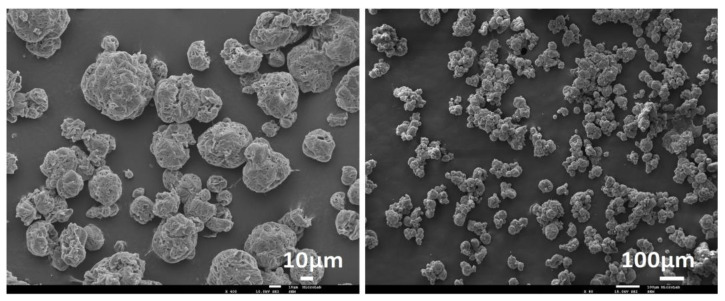
SEM photomicrograph of MCs obtained using a PVA concentration of 4 wt%.

It is noteworthy that, although used for the model development, all the MCs obtained with a PVA concentration of 4 wt% were considered not viable for the current application, as they did not have a spherical shape or, in some cases, significant aggregation. In addition, it was possible to identify some surface holes in the MCs’ shells, which might have compromised its shelf life and the protection of the isocyanate. Figure 5 shows SEM images of MCs produced using 4% PVA, representative of all the MCs obtained with this concentration.

**Figure 6:**
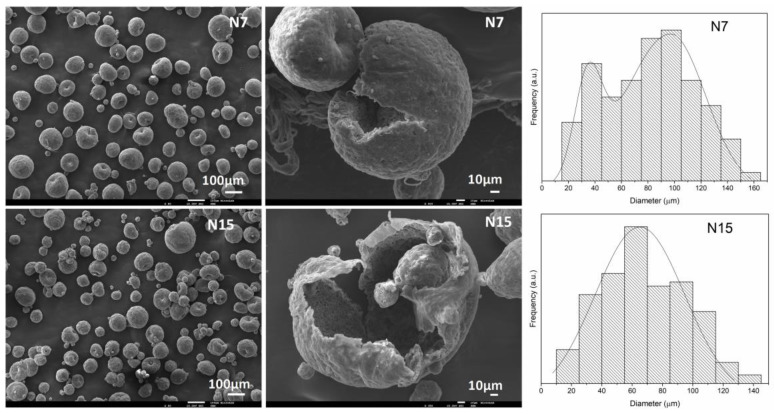
SEM photomicrographs of the MCs from runs N7 and N15 (**left**) and respective size distribution histograms (**right**).

By comparing all the runs proposed by the DoE method and taking into consideration not only the MCs’ average size diameter but also their encapsulation content and shelf life (Table 3), the run N7 (PVAc:2; GAc:3.5; V.r.:39) and run N15 (PVAc:3; GAc:1.75; V.r.:33.5) were the ones that lead to the most suitable MCs. The corresponding SEM images and size distribution histograms are depicted in Figure 6. Run N15 led to MCs with a monomodal size distribution and an average diameter of about 73 µm. N7 led to MCs with a slightly higher average size, of about 90 µm, and a bimodal size distribution but very satisfactory characteristics regarding their encapsulation content and shelf life and were considered as a viable option as well. The average size, respective mode and ratio between the average shell thickness and MCs’ diameter (S/D ratio), for N7, N15, and for the previously reported MCs (I_45 MCs) are listed in Table 6. Both N7 and N15 samples have significantly smaller diameters than the I_45MCs. The size distribution shifted from a trimodal distribution to a bimodal, for N7, and unimodal distribution, for N15, as a result of improved emulsion stability.

**Figure 7:**
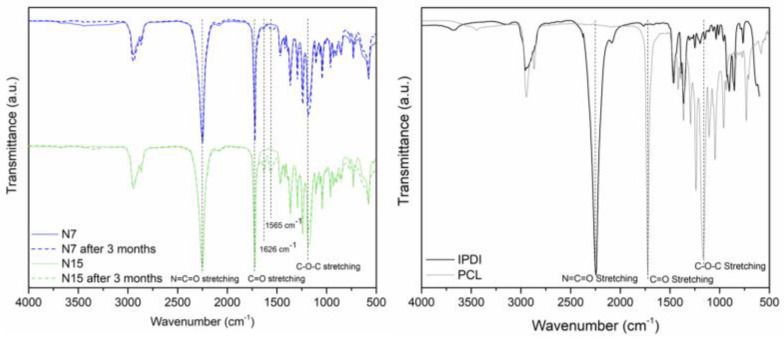
FTIR spectra of the MCs from runs N7 and N15, as prepared and as aged for 3 months at room temperature and 60% of relative humidity (**left**). FTIR spectra of the encapsulated isocyanate and of the shell-forming polymer (**right**).

Figure 7 shows the FTIR spectra of the as-prepared and aged MC, 3 months after its production, as well as the spectra of the encapsulated isocyanate, IPDI, and the shell-forming polymer, PCL. The presence of the peak at 2260 cm^−1^ in all the MC spectra, ascribed to the N=C=O bond stretching vibration, confirms the encapsulation of the unreacted isocyanate. The shell-forming PCL had its most intense peak between 1715–1730 cm^−1^, related to the carbonyl group (C=O) stretching vibration [46,47]. From the identification of this intense peak in the MC spectra, it is possible to confirm the MCs’ shell composition. The bands at 2840–3000 cm^−1^ correspond to the stretching vibrations of the PCL C-H groups within their aliphatic chain, and the ones at 1168 and 1300 cm^−1^ to the C–O–C stretching of the saturated ester groups and to the C–O and C–C groups of main chain, respectively [47]. Typically, the presence of urea is identified from the peaks at 1510 cm^−1^ and 3200–3400 cm^−1^, related to the bending and stretching of the N–H group, as well as from the peak at 1680–1700 cm^−1^, attributed to the urea carbonyl stretching vibration. The absence of the referred peaks, in the as-prepared MC spectra, confirms that no reaction between the isocyanate and the water occurred during the MC production process or, if it did, it was to such a minimal extent that no PUa can be detected.

The peak ascribed to the N=C=O bond stretching vibration, regarding the presence of unreacted isocyanates, maintained its high intensity with negligible changes over a 3-month period. Nevertheless, it was possible to identify new peaks in the aged MCs that were not present in the as-prepared samples, at 1626 and 1565 cm^−1^. The peak at 1626 cm^−1^ was associated with the combined effect of the N–H in-plane bending, the C–N stretching, and the C–C stretching vibrations, which are correlated with urea linkages. The band at 1565 cm^−1^ is associated with in-plane N–H bending, typical of secondary amides [48,49]. These new features in the FTIR spectra reveal the formation of some urea over this period. This is due to the diffusion of water from the air moisture to the MCs’ interior, which consequently reacts with the encapsulated isocyanate, leading to the formation of some PUa moieties. Nonetheless, the high intensity of the peak at 2260 cm^−1^ is an indicator that the polymerization reactions occurred only to a small extent, and the aged MCs were viable as crosslinkers for the application in mind.

The Y value, or relative encapsulation yield (Y), represents an indirect measure of the isocyanate encapsulation efficiency and was used to compare the encapsulation between both syntheses. The obtained Y values, calculated by applying Equation (1), are reported in Table 7. The evaluation of the Y values indicates a slightly higher encapsulation content for the N7_MCs.

**Figure 8:**
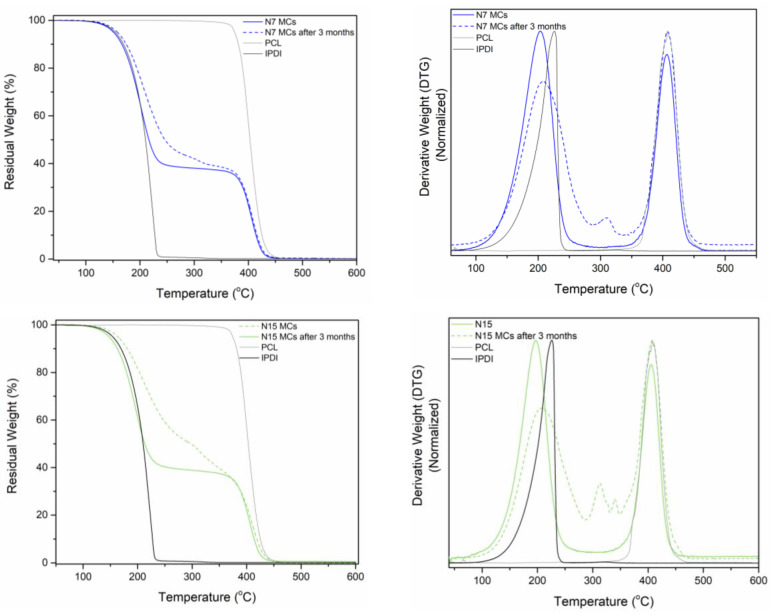
Thermogram of the as-prepared and aged (3 months) N7 MCs and N15 (**left**) and the respective derivative curves (**right**).

The MCs were also evaluated via thermogravimetric analysis to reach conclusions about their encapsulation yield. Figure 8 shows the thermograms of the shell-forming polymer, the encapsulated IPDI, and the N7_MCs and N15_MCs, as prepared and aged for 3 months. The PCL had its degradation peak ranging from ca. 350 to 450 °C, while the IPDI showed its first thermal event between 100 and 240 °C. Both MCs had their first thermal event at around 120 °C, corresponding to the IPDI evaporation, which was used to quantify the encapsulation yield. The isocyanate content of both the N7 and N15_MCs, as prepared and aged, is displayed in Table 7. Both MCs had an IPDI content of around 60 wt%. The DTG of the 3-month-old MCs indicates the loss of some unreacted isocyanate as a variety of low-MW species, oligomers, or low-MW polymeric species, identified from the presence of two peaks, between 280 and 350 °C, not present for the as-prepared MC-derivative thermograms. Indeed, PU/PUa materials have two degradation peaks: the first one between 300 and 370 °C, from the degradation of soft segments, and a second above 370 °C. Comparing the aged I_45MCs thermogram to the first-derivative curves previously reported, both the N7 and N15 aged MCs have a higher content of unreacted isocyanate species, especially the N7 MCs. The N15 MCs seem to have species with a higher degree of polymerization than the N7_MCs. These differences might be due to the variation in the MCs’ S/D ratio. A lower S/D ratio might contribute to a higher encapsulation content, however, it might also be responsible for a lower protection against air moisture diffusion inside the MCs. Although there were variations in the MCs’ composition over the period of 3 months, these were not very significant, with the N7_MCs having a decrease of only 7.4 wt% of its encapsulated content, showing a great resistance to air moisture diffusion.

In the footwear industry, adhesive joints must meet specifications regarding the minimum values obtained in peel strength tests, depending on the type of footwear and the age and gender of the end user. For casual footwear, according with the EN 15307, the peel strength must be higher than 3 N/mm [1,50,51].

**Figure 9:**
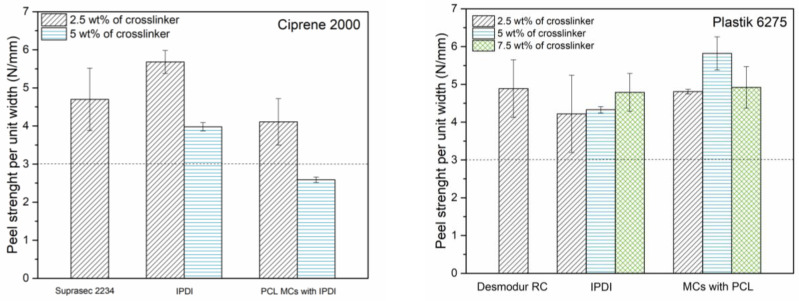
Peel strength test results using adhesive formulations with different concentrations of MCs (run 7), both with Ciprene^®^ 2000 (**left**) and Plastik^®^ 6275 (**right**) as pre-polymers. The dotted line highlights the minimum peel strength value (3 N/mm) required for casual footwear, according with the EN 15307.

**Figure 10:**
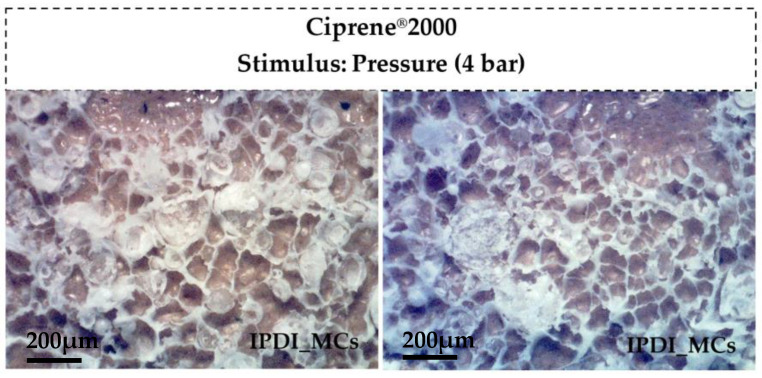
Photographs of the opened bondlines, after the peeling strength tests, using Ciprene^®^ 2000 and PCL MCs.

Figure 9 shows the peel strength test results obtained with non-encapsulated IPDI and PCL MCs (obtained from the run N7), at different concentrations, for both PCP and PU adhesive pre-polymers, Ciprene^®^ 2000 (PCP) and Plastik^®^ 6275 (PU) respectively. Non-encapsulated IPDI led to a high peel strength of the adhesive joints when added at the same concentration as the commercially used crosslinker, indicating its aptness for the application. For Ciprene^®^ 2000, the strength of the adhesive joint seems to decrease for higher isocyanate concentrations. This phenomenon has been already reported in adhesive bonding and is attributed to the excess of free NCO groups in the formulation, which can cause a wider distribution of the crosslinking density with an increase in the adhesive modulus and a decrease of its elongation, affecting the adhesion performance [52,53]. For Ciprene^®^2000, a negative impact of the MCs on the adhesive joints is evident, as the specimens with MCs exhibit lower peel strength values than those with non-encapsulated isocyanates. Even so, with a concentration of 2.5 wt% of MCs, it was possible to obtain a peel strength value above the required 3 N/mm. Figure 10 shows the opened bondlines, after the peeling strength tests, adhered with Ciprene^®^ 2000 and PCL MCs as crosslinkers. Most of the MCs seem to have broken, indicating that the applied pressure was enough to trigger its isocyanate release. Plastik^®^ 6275 is a high-strength bonding pre-polymer, leading to far better results. These specimens resulted in peel strength values well above the required 3 N/mm, with an MC concentration of 5 wt% leading to the most promising results.

Table 8 and Table 9 show the results obtained in the creep test using Ciprene^®^ 2000 (PCP) and Plastik^®^ 6275 (PU) pre-polymers, respectively. The MCs’ performance is compared with the adhesive pre-polymer without crosslinker, with non-encapsulated IPDI, and with the respective benchmark. The use of isocyanates mainly targets a good resistance to creep by promoting an effective crosslinking. The displacement observed for the bondlines prepared with Ciprene^®^ 2000 with encapsulated and non-encapsulated IPDI is similar to that of the benchmark. Regarding Plastik^®^ 6275, it has a high-strength bonding per se, and the main advantage of the crosslinking is to improve the adhesive resistance to temperature. Indeed, it shows almost no displacement of the adhesive bonding even at temperatures as high as 90 °C when either non-encapsulated or encapsulated IPDI is added at 5 wt%. In contrast, in the absence of crosslinker, there is already a full opening at 70 °C. It should be noted that PCL_MCs at 5 wt% result in an adhesive behavior similar to that of the benchmark (Desmodur^®^ RC), with a displacement of less than 1 cm at the higher temperature tested (90 °C). This result indicates the potential for the developed MCs to be used as crosslinkers for highly demanding adhesion joints, using a PU adhesive formulation.

## 4. Conclusions

By using a DoE method, it was possible to develop a model to predict the optimum values for three pre-defined variables, known to influence MCs’ average diameter. The variables defined to control the average size of the MCs were the GA and PVA concentrations in the W phase, and the O phase volume on the total emulsion system, based on their well-known influence on emulsion stability. PVA concentration was found to play the biggest role in the MCs’ size; on the other hand, the O phase volume was the parameter which influenced it the least. By applying the developed model, it was possible to decrease the MCs’ average size from 326 µm to 70 μm. Improvements both in the encapsulation yield and protection against air moisture diffusion were achieved, occurring only a minimal loss, between 7.4 wt% to 13.5 wt%, of the encapsulated content over a period of 3 months. Considering the MCs average size diameter, its encapsulation content and shelf life, the conditions used for the run N7 (PVAc:2; GAc:3.5; V.r.:39) and the run N15 (PVAc:3; GAc:1.75; V.r.:33.5) were the ones leading to the most suitable MCs.

The PCL MCs were able to respond either to one or both stimuli (pressure and temperature) applied in the adhesive joint preparation. The MCs were able to release their content during the adhesion process and act as crosslinkers, which was confirmed both by the increased peeling strength and temperature resistance, compared with the non-crosslinked pre-polymer. The adhesive formulation obtained with the Plastik^®^ 6275 pre-polymer (PU based) and 5 wt% of PCL MCs lead to adhesive joints which responded to both the peeling strength and creep tests similarly to those prepared with the benchmark crosslinker, showing the potential of these MCs as crosslinking agents for high performance adhesive formulations. The optimized MCs can be further used to other applications, such as self-healing approaches and smart materials.

## Figures and Tables

**Table 1:** Experimental range and level of variables (input in the MODDE^®^ Pro 13 software).

Independent Variables	Abbreviation	Factors	Coded Levels		
			−1	0	1
PVA concentration % (*w*/*w*)	PVAc	$x_{1}$	2	3	4
GA concentration % (*w*/*w*)	GAc	$x_{2}$	0	1.75	3.5
O phase volume % (*v*/*v*)	V.r.	$x_{3}$	28	33.5	39

**Table 2:** The experimental conditions run to perform the DoE study, given by the MODDE^®^ Pro 13 software.

Experimental Run	Experimental Parameters			Response
	W Phase		V.r. (% *w*/*w*)	MCs Size (µm)
	PVAc (% *w*/*w*)	GAc (% *w*/*w*)		
N1	2	0	28	Y_1_
N2	4	0	28	Y_2_
N3	2	3.5	28	Y_3_
N4	4	3.5	28	Y_4_
N5	2	0	39	Y_5_
N6	4	0	39	Y_6_
N7	2	3.5	39	Y_7_
N8	4	3.5	39	Y_8_
N9	2	1.75	33.5	Y_9_
N10	4	1.75	33.5	Y_10_
N11	3	0	33.5	Y_11_
N12	3	3.5	33.5	Y_12_
N13	3	1.75	28	Y_13_
N14	3	1.75	39	Y_14_
N15	3	1.75	33.5	Y_15_
N16	3	1.75	33.5	Y_16_
N17	3	1.75	33.5	Y_17_

**Table 3:** The experimental conditions runs and respective experimental results.

Experimental Run	Experimental Parameters			Response	Encapsulated Isocyanate (wt%) (As-Prepared MCs) *	Encapsulated Isocyanate (wt%) (after 3 Months) *
	PVAc (% *w*/*w*)	GAc (% *w*/*w*)	V.r. (% *w*/*w*)	MCs Size (µm)		
N1	2	0	28	155.1 ± 74.5	58.3	41.4
N2	4	0	28	41.4 ± 15.8	57.9	40.6
N3	2	3.5	28	109.1 ± 33.1	58.8	48.7
N4	4	3.5	28	46.5 ± 14.2	57.4	38.9
N5	2	0	39	172.6 ± 83.6	59.6	52.3
N6	4	0	39	82.8 ± 23	57.7	41.2
N7	2	3.5	39	93.7 ± 32.1	60.6	53.1
N8	4	3.5	39	91.8 ± 46	55.1	40.2
N9	2	1.75	33.5	140.5 ± 79.8	58.2	41.8
N10	4	1.75	33.5	65.5 ± 21.5	56.6	36.7
N11	3	0	33.5	104.2 ± 48.6	57.1	42.3
N12	3	3.5	33.5	70.8 ± 25.1	58.5	47.1
N13	3	1.75	28	70.2 ± 32.8	58.5	45.3
N14	3	1.75	39	109.4 ± 29.9	58.2	45.8
N15	3	1.75	33.5	76.6 ± 31.2	58.2	44.8
N16	3	1.75	33.5	81.2 ± 25.2	56.7	46.1
N17	3	1.75	33.5	130.1 ± 92.4	59.1	48.4

* Results obtained via thermogravimetric analysis.

**Table 4:** Results of the multiple regression analysis.

Regression Coefficients	R^2^	R^2^ Adj.	Q^2^	RDS
Coefficient Values	0.973	0.955	0.867	7.809

**Table 5:** Experimental runs for validation of the model and respective predicted and experimental results.

Experimental Run	PVAc (%)	GAc (%)	V.r. (%)	MCs Average Size (µm)	
				Predicted	Experimental
N18	3	1.5	28	58.6–93.4	90.3 ± 25.3
N19	2.8	2	33.5	66.4–88.1	80.2 ± 20.9

**Table 6:** N7, N15 and I_45MCs average size, size distribution mode and S/D ratio.

MCs’ Acronym	Average Size	Mode	S/D Ratio
N7	93.7 ± 34.1	38.7, 98.5	0.38, 0.15
N15	72.9 ± 32.2	62.8	0.087
I_45MCs	326.4 ± 157.3	264, 416 and 637	0.11, 0.071 and 0.046

**Table 7:** N7_MCs’ and N15_MCs’ relative encapsulation yield (Y value) and mass loss (%) of encapsulated IPDI for each experiment.

MCs’ Acronym	Relative Encapsulation Yield (Y), from FTIR	Encapsulated Isocyanate (wt%), from TGA	Encapsulated Isocyanate in the 3 Months Aged MCs (wt%), from TGA	% of Isocyanate Loss after 3 Months
N7 MCs	3.19	60.6	53.1	7.4
N15 MCs	2.67	58.2	44.8	13.5

**Table 8:** Results obtained in the creep tests for substrates adhered with a pre-polymer and adhesive formulations containing Suprasec^®^2234 and IPDI as crosslinkers, as well as PCL MCs containing the last, using Ciprene^®^2000 as pre-polymer.

Crosslinker	Percentage in the Pre-Polymer (wt%)	d (cm) 60 °C	d (cm) 70 °C	d (cm) 80 °C	d (cm) 90 °C
-	-	FO	FO	FO	FO
Suprasec^®^ 2234	2.5	0.35	1.05	3.35	FO
IPDI	5	0.65	2.75	3.25	FO
PCL_MCs	2.5	1	1.93	3.95	FO
	5	0.83	2.30	3.40	FO

**Table 9:** Results obtained in the creep tests for substrates adhered with a pre-polymer and adhesive formulations containing Desmodur^®^ RC and IPDI as crosslinkers, as well as PCL MCs containing the last, using Plastik^®^6275 as pre-polymer.

Crosslinker	Percentage in the Pre-Polymer (wt%)	d (cm) 60°C	d (cm) 70°C	d (cm) 80°C	d (cm) 90°C
-	-	1	FO	FO	FO
Desmodur^®^ RC	2.5	0	0	0	0
IPDI	2.5	0.3	0.96	FO	FO
	5	0	0	0	1.43
PCL_MCs	2.5	0	0.33	2.07	FO
	5	0	0.2	0.27	0.93

## Data Availability

The data presented in this study are available in the present article.
